# *“An eye-opener:”* a qualitative study of a liberal arts approach to medical education

**DOI:** 10.1186/s12909-025-07157-z

**Published:** 2025-04-25

**Authors:** Abebe Bekele, Denis Regnier, Claire O. Swedberg, Eden Abate Lemu, Christelle Uwantege Giraneza, Elizabeth H. Bradley

**Affiliations:** 1 University of Global Health Equity, Butaro, Rwanda; 2 Vassar College, 124 Raymond Ave, Poughkeepsie, NY USA

**Keywords:** Medical education, Africa, Humanities, Social sciences, Liberal arts

## Abstract

**Background:**

Medical educators have underscored the need for medical students to study the larger sociocultural and economic forces that influence health rather than simply basic and clinical sciences; however, previous studies have not evaluated the impact of implementing a full-scale liberal arts approach to medical education. Such a model has been implemented at the University of Global Health Equity (UGHE) in Rwanda, and we sought to evaluate the student experience.

**Methods:**

We used a qualitative study with a grounded theory approach with in-depth interviews of MBBS students at UGHE. Interviews were conducted by members of the researcher team unknown to MBBS students using a semi-structured discussion guide; interviews continued until the point of theoretical saturation, and we used the constant comparison method of qualitative data analysis to understand recurrent themes.

**Results:**

Participants (*n* = 18) were evenly split between male and female; 66.7% were from Rwanda and 33.3% were international students. Participants had completed the liberal arts component of the curriculum, which was given in the first 6 months of the MBBS. Recurrent themes emerged in four broad areas pertaining to what the liberal arts approach was and its impact on students, their peer groups, and their perceived clinical capability. The four recurrent themes were: (1) the liberal arts experience encompassed a unique approach to class content, pedagogy, and culture, (2) it widened student perspectives, (3) it strengthened peer relations and teamwork skills, and (4) students believed it improved their clinical capability.

**Conclusions:**

Medical students reported a profound effect of including a liberal arts approach in the medical undergraduate curriculum as delivered at UGHE. With increased accessibility to online education, it has never been more important to examine and support the humanization of education–particularly for medical students who wish to tackle global health equity. A liberal arts approach may offer a path forward.

**Supplementary Information:**

The online version contains supplementary material available at 10.1186/s12909-025-07157-z.

## Background

Efforts to expand and transform African medical education in recent decades have been many [1–4]. These have included the opening of new medical schools, the use of increasing enrollment in existing medical schools, e-learning, curricula integration, expansion of simulation laboratories, and engaging community-based learning more thoroughly [2, 3, 5–8]. Many of these efforts have been made in response to the traditional pedagogy followed in most medical schools, which relies upon instructor-centered lectures and memorization-based examinations, potentially preventing students from actively engaging in their learning [9].

At the same time, the need for medical students to study the larger sociocultural, economic, and political forces that influence health–what might be called the domain of social medicine–rather than simply basic and clinical sciences has been previously underscored [10, 11]. Such thinking argues that physicians who understand the social, psychological, behavioral, and environmental determinants of health will be more equipped to influence not only their patients’ health but the broader goals of population health and health equity. In the US, the medical admissions exam has been amended, precisely to better recognize the importance of social sciences and humanities as key aspects of developing effective physicians [11]. Although multiple studies examine the effect of introducing social sciences and humanities courses into medical education [12–18], previous research has not evaluated the impact of implementing a full-scale liberal arts approach to medical education in which the curriculum reflects a range of humanities and social sciences, faculty use inquiry-based pedagogy, and the classroom culture is designed to inspire deep engagement by students.

Accordingly, we sought to explore student experiences with a liberal arts approach to medical education with the goal of understanding its capacity to engage medical students and foster new skills. We conducted the study at the University of Global Health Equity (UGHE) in Butaro, Rwanda. UGHE was founded in 2015 by Dr. Paul Farmer and Partners in Health (PIH) in collaboration with the Rwandan Ministry of Health to develop in medical trainees and subsequent physicians a greater understanding and commitment to social medicine, particularly for caring for low-income and marginalized patients and populations. To meet our study objective, we conducted a qualitative, in-depth interview study using a grounded theory approach [19–21] of students in all years of the curriculum. Findings may be useful to medical educators and policymakers who are working to expand access to innovative medical education and improve global health equity.

## Methods

### Setting: liberal arts approach

A liberal arts approach to medical education has been previously described [22], which encourages an engagement between students, their instructors, and the curricula which fosters critical thinking, creative problem-solving, and an understanding of broader social forces that shape the livelihoods and health outcomes of individuals and populations. This approach aims to equip future physicians with the tools to conduct insightful analysis in their careers and prepare them not only to be excellent physicians for their patients but also to understand and address the social determinants of health and health equity.

At UGHE, the liberal arts are implemented in the first 6 months of the MBBS program; students are full-time living on campus in dormitories during this time and they take a set of 12 classes. The classes have included, for example, critical thinking and scientific reasoning, academic writing, health psychology, African history and political economy, health information technology, and communication. This first part of the MBBS curriculum is taught using inquiry-based, experiential pedagogy as much as possible, in an institutional culture that prioritizes equity. Student assessment is done using a rubric shown on the syllabus for each class typically using graded performance on a combination of papers, problem sets, presentations, and class participation.

### Reflexivity statement

The authors include faculty (AB, DR, EHB) who teach in the UGHE program, and two (AB, DR) have administrative roles in the university. The other authors (COS, EAL, CUG) were unaffiliated with the MBBS program although they had recently completed a master’s program at UGHE. These relationships helped provide insights and access to information and may have influenced the course of the project. Authors who had teaching or administrative roles in the MBBS program were not involved with interviewing, and all student data was anonymized in transcription to limit bias based on author relationships to the institution or participants. Together the team reflected periodically on bias that may emerge based on our roles, and specifically, we sought disconfirming evidence to limit groupthink and listen to negative feedback objectively.

### Ethics approval and consent to participate

Prior to beginning the study, the research protocol and informed consent procedures and form were reviewed and approved by the Institutional Review Board (IRB) Human Subjects Review by the University of Global Health Equity (UGHE) and adhered to the Declaration of Helsinki. All subjects provided informed consent–either in written or oral form, which was deemed appropriate by the IRB given the negligible risks associated with participation.

### Study Design

We undertook a qualitative, in-depth interview study [23, 24] using a grounded theory approach [21] to explore in rich detail students’ experiences in the liberal arts components of the UGHE curriculum. The grounded theory approach [21] was deemed appropriate because little is known about medical students experience with a liberal arts approach to medical education, particularly in low-income settings and in Africa, and we sought to develop theory or conceptual categories that might characterize the potential impact of such a curriculum on future physicians. In-depth interviewing was used to generate insights, including hypotheses about students’ experiences, which may be tested in subsequent quantitative studies. Our team of researchers had previous and current relationships with UGHE, including three former students of a different educational program at UGHE, a collaborating professor who also directs a liberal arts college in the US, and the dean of UGHE.

### Sampling

As is standard in qualitative research, we used purposeful sampling [25] to attain a diverse set of study participants. First, we randomized all registered UGHE MBBS students (*n* = 215) over five years of the program. Recognizing that year in the program, gender, and international status (versus Rwandan nationality) may influence students’ MBBS experience, we sought to ensure the sample was adequately diverse across these characteristics. We selected students in random order, skipping individuals who had a year, gender, and international status profile of other students already selected for the study. We continued to select and interview students until we achieved theoretical saturation [21], i.e., until no new concepts emerged from successive interviews. This occurred after 18 interviews.

### Data Collection

After obtaining informed consent, we conducted in-depth interviews in person when possible (22%) and virtually (78%) when needed. Interviews were conducted by members of the research team (COS, EAL, CUG) who were unknown to the MBBS students using a discussion guide made up of open-ended, *grand tour* questions [26] and including probes in areas of potential interest. The discussion guide (Additional file 1) was developed specifically for this study and piloted with four students, who were then excluded from being eligible for the full study. The discussion guide was revised based on the pilot to improve clarity and then then used consistently with all study participants. After consent for recording, interviews were audiotaped and professionally transcribed using Rev. Interviews averaged about 30 min in length, and transcriptions were checked against audiotapes for accuracy and corrected as needed.

### Data Analysis

We employed the constant comparative method of qualitative data analysis [21, 27, 28] to analyze the interview data. The coding process was undertaken by a multidisciplinary research team with diverse backgrounds (COS, EAL, CUG, EHB) and began with reading early transcripts for general understanding and inductive development of the code sheet. Independently, the four coders identified chunks of data that illustrated a concept and affixed a label (subsequently a code) to that chunk of data, constantly comparing with previous chunks of data that had been labeled and coded similarly to refine the meaning and boundaries of each concept. After coding each transcript independently, the four coders met to resolve disagreements through negotiated consensus. With each review, codes and the code sheet were revised (e.g., added new codes, combined existing codes under a broader concept, provided greater detail to the meaning of the code). This process continued until we arrived at a final code sheet with descriptions and illustrations of each concept apparent in the interviews. Using the final code sheet (comprising 42 codes), two coders re-coded all transcripts; although we did not calculate inter-rater reliability, each difference was reviewed and resolved through negotiated consensus–in some cases by reconvening the four coders and in other cases by the two final coders. We kept an audit trail documenting analytic decisions throughout the process, and we used the qualitative data analysis software Dedoose to facilitate data retrieval and analysis.

## Results

### Participants

Participants (*n* = 18) were diverse, representing 50% male and 50% female medical students. A total of 66.7% were from Rwanda while 33.3% were international students at UGHE; one third were pre-clinical (the first two years of the curriculum) and two-thirds were clinical (in last four years of the MBBS). All participants had completed the liberal arts component of the curriculum, which is given in the first 6 months of the MBBS.

### Overall experience

Although many were surprised (some were even disappointed) when they first arrived at UGHE that they did not immediately study basic science or clinical science classes, all participants reflected positively on the liberal arts portion of the curriculum. Participants reported that, over the first months of the curriculum, their initial confusion gave way to appreciation and even inspiration about the academic experience they were having. Some suggested the phase be shorter while others–particularly students who had the liberal arts phase online due to COVID–expressed wishing it had been more immersive. In general, however, participants expressed high satisfaction with this part of the medical curriculum.

### Recurrent themes

Recurrent themes emerged in four broad areas pertaining to both what the liberal arts approach was and its impact on the students, their peer groups, and their clinical capability. The four themes were: (1) the liberal arts experience encompassed a unique approach to class content, pedagogy, and culture, (2) it widened student perspectives, (3) it strengthened peer relations and teamwork skills, and (4) students believed it improved their clinical capability (see Table 1 for themes and coded concepts within themes). Below, we describe each of these, supported by illustrative quotations from the participants.

**Table 1:** Themes and coded concepts within themes

Themes	Coded concepts within themes
Overall experience	Surprise, adjustment, relevance, institutional support, language, student voice
The liberal arts experience encompassed a unique approach to class content, pedagogy, and culture	Liberal arts, critical thinking, tackle root causes of disease, teaching style, classroom culture, campus culture, writing/presentation skills, computer skills
It widened student perspectives	Broader perspective, eye-opening, gender
It strengthened peer relations and teamwork skills	Interpersonal and intergroup relationships, teamwork, collaboration, mental health
Students believed it improved their clinical capability	Connections to clinical work; community engaged learning, advocacy

#### Theme #1. Liberal arts experience: class content, pedagogy, and culture

In describing the liberal arts part of their curriculum, participants remarked on the breadth of the course content, the teaching style of the faculty, and the classroom culture. They noted that the diversity of topics and readings they were exposed to let them “see the bigger picture” concerning the socioeconomic, political, cultural, and historical roots of health and disease. For instance, participants said:

The term liberal arts means…I can say liberal arts, it seeks to liberate the mind. It includes, for example, critical thinking and all those courses that we study. They help us to think critically, to analyze different situations, and to see the real world with a critical eye. (ID 2, 1st year male Rwandan student).

[We were] speaking of political economy and geopolitics, looking at how…the international monetary fund, the World Bank, all this in one way or another affects countries and sometimes poverty is perpetrated [by] all of this…It was kind of an eye opener for me to look at medicine and health and public health from a different angle. Not necessarily in the hospital but also widen my horizons and look beyond that. So that was the most important thing that I really got from [the liberal arts classes]. (ID 20, 5th year male Rwandan student).

Participants expressed both surprise and enjoyment of the teaching style as distinct from what they had previously experienced, either in secondary school or at another higher education institution. In particular, the accessibility of the faculty, the creative activities in class (e.g., cross-dressing day to understand gender dynamics), the assigning of group projects, and the fostering of interactive discussions among peers and with the faculty in the classroom (even about controversial topics such as sex work, cultural taboos, or mental health) were highlighted by participants as novel aspects of the liberal arts curricular time.

[At UGHE], I was introduced to a new [kind of] class where you could question everything, where you had to ask questions, where you had a dynamic class, [with] people being so expressive. I think it has shaped me in a way that I don’t feel so intimidated to share what I think, even if it’s controversial. (ID 16, 5th year male Rwandan student).

Participants also reflected on the classroom culture, which was referred to as: “shocking but very good.” The shocking nature referred to how engaged faculty were and how much participation and critical thinking by students was not only allowed but expected. At the same time, participants described feeling safe to take intellectual risks, being comfortable with each other, and learning from their peers–not just from the instructor. Participants used adjectives such as free, flexible, dynamic, and expressive to describe the classroom culture. Furthermore, they indicated that broader campus culture was marked by faculty caring not only about students’ academic growth but also about their personal growth, as well as the value of equity. Following are a selection of illustrative quotations concerning the classroom and campus culture in which the liberal arts curriculum was experienced:

Being in a class where everyone was expressive and everyone was encouraged to give ideas was so helpful. Going with your teachers to lunch and discussing other things out of class, and also they’re interested in your academic growth, but also in your personal growth. Also having office hours that we had every Wednesday afternoon where you could have a one-on-one interaction with the teacher that was new. And you could send an email to your professor of your essay to give you feedback and you could easily get feedback. (ID 16, 5th year male Rwandan student).

Me and my classmates grew up thinking that whatever the teacher says stands, even if I disagree, I’m going to keep it to myself. But the [liberal arts] teachers kept encouraging us to speak up; what do you think? What are your personal thoughts on this? And that encouraged us to even think critically because if you’re in a class and everything is about cramming. I’ll take what I’m told, I’ll accept it. But when someone shows interest in your [input], it actually encourages you to think. And then from thinking, you also get an opportunity to share your ideas. (ID 7, 1st year female Rwandan student).

Furthermore, participants indicated that the classroom culture reflected and reinforced the university’s commitment to equity. Participants noted that “high-ranking people at UGHE often dined with students” and they witnessed equity in how students, faculty, administrators, and even the Board of UGHE interacted informally on campus. Although one participant noted the desire for a greater student voice on campus, many highlighted the experience of social equity, called “symmetry” by one participant.

I was surprised to see high-ranked people, who are known in the world, people who had great CVs, people who did great things in the world–you would sit with them…and they would actually like to talk to you. I was surprised by that. Well, primary and high school teachers, they all have their own dining. They have their own living areas. You don’t talk to them, you respect them. You almost have to bow down and everything. But then there were people [here] on the board, people whose names I can’t even pronounce. But they’re like, “come join us. We want to speak to you.” There’s that kind of “symmetry.” (ID 4, 5th year female Rwandan student).

#### Theme #2. Widening perspectives and critical thinking

A recurrent theme was about the impact of the liberal arts on expanding a student’s perspective on many issues. Participants described the courses as opening them up to a wider worldview, particularly about the social dimensions of health and disease. They said the experience was “eye-opening” and added to their “open-mindedness” as they felt themselves growing less biased as they understood the greater diversity of experience–particularly as it related to poverty and marginalization. Additionally, participants offered that they had grown in self-awareness, understanding their privilege as medical students more clearly. Some described developing more empathy and that they now had “stopped playing the blame game,” as they recognized that too often people were blamed for their illness, suffering, or poverty. Paradoxically, participants reflected that they had grown in confidence and also in humility. In the words of participants:

##### Widening perspective

For me personally, the liberal arts were courses that showed me a very big picture of how people live in the real context. You can encounter someone, and you’re thinking science, science, science. But liberal arts give you that dynamic of thinking about the person in a very large context and consider emotions, their social context of living, and all that. As a medical student, this also helps me while I’m encountering a patient–knowing the very big picture of the person. (ID 19, 4th year female Rwandan student).

##### Confidence

I guess I could say I gained confidence because I always was asking questions, even to the point it was annoying for others. But no, I guess I gained that sense of comfort where it’s not a negative thing to be curious, question anything and everything around you, and if there’s an answer, you’ll get the answer. That’s what I mostly gained. (ID 11, 3rd year female International student).

##### Humility and Empathy

So, it’s like we got to understand where people are coming from. I’m a medical practitioner and then a person comes in. They’re not so clean with dirty feet and all that. You have to understand where they’re coming from. Sometimes we get judge-y. We think people are not taking care of themselves, when actually it’s where they’re coming from. It might be that they don’t have water and all that. So, I think that [the liberal arts experience] makes us more empathetic. (ID 26, 1st year female international student).

The critical thinking aspects of the curriculum were appreciated as participants described that they had become skeptical of superficial explanations and instead understood how important asking why and getting to the root causes was for effective problem solving.

In terms of critical thinking…I was given another lens to look at things from a different angle. What about if you thought about it this way? So, my critical thinking skills improved very much. I didn’t know that you could even see things in a different way. Whenever you say something is bad, try to put yourself into their shoes. So those are the kind of things that we gained. Knowledge in terms of critical thinking, knowledge in terms of social injustices in the medical world, and I mean inspiration [from the] lectures and the classes. (ID 14, 3rd year male international student).

I think liberal arts changed the way that I think, not even just about my classes, but in terms of what I think as a human being. So liberal arts encourages you to question, think about stuff inquisitively, don’t just accept everything that you are told. Do your own research, read more, ask yourself questions. Ask questions to the people around you. And the way that impacted me is that it changed the way I overall think about things. (ID 7, 1st year female Rwandan student).

#### Theme #3. Strengthening peer relationships and teamwork

The liberal arts phase of the curriculum was described as strengthening peer relationships and teamwork. Participants expressed how they had grown in their respect for their peers’ knowledge and inputs, how their interpersonal communication skills had improved, and how the pedagogy helped them build collaboration skills. Importantly, participants viewed these skills as integral to their later clinical practices and teamwork in the hospital. In the words of participants:

What I gained was about interpersonal communication because the activities were done in groups. So, I gained how to connect or how to relate with others and how to work together with others as a team. (ID 2, 1st year male Rwandan student).

It was so surprising to find how relevant [the liberal arts experience] can be in application when you get to the hospital–how you’re going to need the ways of communication you’ve learned, the kind of decision-making you’re going to use. So it was very surprising for me to find how very, very closely related [the liberal arts curriculum] and my clinical experience are. (ID8, 3rd year female Rwandan student).

#### Theme #4. Improving clinical capability

Last and perhaps most importantly, participants who had advanced to the clinical part of their MBBS training discussed how the liberal arts experience had improved their clinical capability with patients and the community. Participants said:

*Caring for a patient with chronic headaches*: But if you go further, you can find that many factors are related to that headache. Maybe it’s not just because of migraines; maybe it’s because of the way her husband treats her. Maybe it’s something deeper. You can find that it’s something with other roots. I think [that’s where] we got to learn about social determinants of health. And we really were taught how to treat and see patients as a whole and try to really try to tackle the real cause of the problems. Because she might come in with diarrhea, but then it’s not just her. Maybe it’s the whole community, maybe it’s an issue with the water there. Maybe they don’t even have water. Maybe it’s something that started from the administration, from the local leaders. It’s an issue that maybe that is way deeper than what you think the patient has right now. (ID 4, 5th year female Rwandan student).

*Caring for a patient with sarcoidosis*: When you get into clinicals, most of us are just looking at the disease aspect and the science of it all. But I try to recall back to my classes that I learned in the [liberal arts], and think okay, this guy, he has sarcoidosis secondary to what? He’s a miner. So, I got it from silica dust within the mines. And so, we can’t just tell the guy, okay, this is a permanent disease, we’re just going to have to give him O2 support for the rest of his life…We also have to think about how we are going to tell him he can’t go back to work in the mines? How can we ensure that he can afford the medication? How can we make it convenient for him to go to a nearby health center that can provide these services rather than take a long journey over to the hospital and all that stuff? (ID 11, 3rd year female international student).

The liberal arts content of the MBBS curriculum allowed medical students when in the hospital or working in the community to think more creatively about ways to approach patients and communities about health challenges, tackle the root causes of disease, and advocate for marginalized patients and communities needs in the face of inequitable and unjust economic, sociocultural, and political structures. Reflecting on what they had gained from the liberal arts curriculum, one participant said:

The connections I made in courses about social justice, gender, and medical anthropology…introduced to us some of the biggest challenges our patients face in the community. They also introduced to us also the most prevalent challenges in global health. The kind of connection I find here with the clinical experience [is] when we treat patients, they encourage us to see patients not as a disease but as a human being. And we are trained to see a disease as a result of multiple social forces that act against the patient. (ID 13, 4th year male international student).

## Discussion

Medical students described the liberal arts approach as having a profound effect on their medical school experience at UGHE. Students experienced the breadth of the course content, the teaching style, and the culture in which the curriculum is delivered to be eye-opening, engaging, and relevant to their subsequent clinical practice. The impact of the educational intervention was described as shifting students’ perspectives to understand the broader causes and context of disease and health inequity, enhancing self-awareness and humility, and strengthening empathy for patients and their communities. Interestingly, older students were particularly articulate about the relevance of their liberal arts courses to their clinical practice and current commitments to social medicine and promoting health equity.

Although students expressed being highly satisfied with the liberal arts approach, they identified shortcomings as well. These included attending to student mental health more thoroughly, sustaining nascent avenues for students’ voice in the governance of the institution, and recognizing language challenges as students come from different countries. Furthermore, replicating the UGHE model may be challenging. For instance, finding local faculty who are equipped to teach both the broader set of topics and use inquiry-based pedagogy can be difficult; however, future efforts may be possible with global collaborations and potential variations on the UGHE approach. The concepts identified in this research can form the basis of a range of educational innovations that may be more efficient and still impart the knowledge and inspiration to expand the influence and positive impact of physicians in social medicine.

This research extends the existing literature on humanities in medical education, which has largely focused on medical training in high-income countries and evaluated efforts to add a single humanities course or a humanities module to the more traditional medical curriculum [14–16, 29]. These extant studies have reported mostly positive effects on empathy, professionalism, self-care, tolerance for ambiguity, and reduced burnout [14–16, 29]. Studies from low- and middle-income settings [30, 31] have reported mixed results with concerns related to relevance [30], limited student understanding of social accountability [31], limited resources [15] and the potential for schools to fall into the “content trap,” looking to specific courses rather than a broader approach to the medical school experience to confer humanistic competencies [32]. In contrast, our study finds consistently positive outcomes, as students recognized not only the course content but also the pedagogical style and institutional culture in which the courses were delivered as integral to their learning and development. Furthermore, although the intervention requires attention to building faculty teaching capacity, it is relatively inexpensive as it does not require new laboratory equipment, sophisticated imaging, or other resource-intensive infrastructure.

Medical educators may use these findings to inspire curricular and pedagogical reviews of medical education in other contexts, particularly where physicians are expected to encompass broader perspectives on medicine that include the social determinants of health and health equity. A heartening piece of evidence from this study was the enthusiastic engagement and commitment to medicine fostered in medical students through the liberal arts approach to their educational experience. This enhanced commitment to the profession coupled with the strengthened peer relationships and teamwork may have positive longer-term effects at the health system level although future studies are needed to understand those potential effects.

Our findings should be interpreted in light of some limitations. First, the study was completed at a single university equipped with multiple academic and community partnerships to support the mission of global health equity and with a likely selection of students interested in social medicine. The university has substantial institutional support, resources, and commitment; hence results may differ in other institutions. Second, we used a qualitative approach, which limits our ability to infer causality or generalize from our findings. Nevertheless, we employed rigorous methods as recommended by experts in qualitative methods to enhance trustworthiness [19, 24, 33, 34] including the use of a standardized, pretested discussion guide, multiple interviewers to help with interpretation, audiotaping of interviews, independent and quality-assured transcription of the audiotapes, a coding team with diverse backgrounds and perspectives, consistent application of the coding scheme, resolution of coding disagreements by negotiated consensus, and use of an audit trail to document analytical decisions. Last, the results reflect students’ experience; we were not able to corroborate their perception or triangulate our findings with views from their medical supervisors or patients, which would strengthen the trustworthiness and warrants future study. Nor did we have UGHE alumni perspectives as the first MBBS class had not yet graduated. Nevertheless, the students–over diverse years and experience in medical school–provided concrete examples and narratives that enhanced the credibility and compelling nature of their experiences.

In this moment of increased accessibility to online education, it has never been more important to examine and support the humanization of education–particularly for medical students who wish to tackle global health equity. Futurists [35] tell us that as technology grows in its influence, the characteristics of curiosity, creativity, and compassion–and, we would add, sensitivity to issues of social justice and equity–will be what distinguishes human-centered work and what will be valued over time. Based on our findings, these characteristics and skills along with a deep commitment to health equity for patients and their community can be nurtured and strengthened through an in-person liberal arts approach to coursework, pedagogy, and institutional culture throughout the medical education experience.

## Discussion

All research procedures, including the informed consent processes, were reviewed and approved by the Institutional Review Board (IRB) Human Subjects Review of the University of Global Health Equity (this is its full name).

## Electronic supplementary material

Below is the link to the electronic supplementary material.

Supplementary Material 1

## Data Availability

No datasets were generated or analysed during the current study.
