# Supplementary material for: “An eye-opener:” a qualitative study of a liberal arts approach to medical education
Source: BMC Med Educ. 2025 Apr 25;25:610. doi: 10.1186/s12909-025-07157-z (PMC12023582; doi:10.1186/s12909-025-07157-z)
Supplement: Supplementary file 1 — Supplementary Material 1 [file 12909_2025_7157_MOESM1_ESM.docx]

**Figure 1: Discussion Guide**

1. How did you first hear about the MBBS at UGHE and tell us the story of how you decided to apply?

2. Can you describe your experience in the first six months of the MBBS (sometimes called the prep phase in which you experienced a range of social science and humanities courses)?

3. What have you been taught about “liberal arts?” What does that term mean to you?

4. Tell us, in your own words, what did you gain from the prep phase? (if they do not address these concepts spontaneously, then probe: content of classes, teaching style emphasis on critical thinking, connections between instructors and students, classroom culture, and campus culture)?

5. *For participants in cohorts who have begun clinical:* What connections, if any, have you seen between the humanities and social science part (i.e., prep phase, liberal arts) of your education and your clinical experience? (Probe about connections to the community, connections to patients, critical thinking in the clinical and field experiences)

6. What has surprised you about your experiences of UGHE?

7. We are trying to understand students’ experience, particularly related to the liberal arts components (prep phase) of the MBBS. Given this, is there anything else we should have asked you to better understand your experience? If you have anything to add, emphasize, clarify or a question you believe we should have asked you, please take this opportunity and share with us.
